# Supplementary material for: Reservoir computing based on a silicon microring and time multiplexing for binary and analog operations
Source: Sci Rep. 2021 Aug 2;11:15642. doi: 10.1038/s41598-021-94952-5 (PMC8329232; doi:10.1038/s41598-021-94952-5)
Supplement: Supplementary file 1 — Supplementary material 1 (pdf 267 KB) [file 41598_2021_94952_MOESM1_ESM.pdf]

Reservoir computing based on a silicon microring and time multiplexing for binary and analog operations: supplemental document

Massimo Borghi*, Stefano Biasi, and Lorenzo Pavesi
Nanoscience laboratory, Department of Physics, University of Trento, Via Sommarive 14, 38123, Trento, Italy and
**corresponding author: massimo.borghi@unitn.it*

A. DERIVATION OF THE RESERVOIR DYNAMICAL EQUATION

Here we derive Eq.(1) of the main text, which describes the time evolution of the probe at the output of the Drop port of the resonator. The starting point is the equation governing the free carrier dynamics, which is given by [1]:

$$\frac{d\Delta N}{dt} = -\frac{\Delta N}{\tau_{\text{fc}}} + g_{\text{tpa}} U^2, \tag{1}$$

where g_{tpa} is the free carrier generation rate per unit energy induced by TPA and U is the internal energy of the resonator. Equation (1) has the formal solution:

$$\Delta N(t) = g_{\text{tpa}} \int_{-\infty}^{t} e^{-\frac{t-\xi}{\tau_{\text{fc}}}} U^2(\xi) d\xi. \tag{2}$$

The total energy is given by $U = U_p + U_{pr}$, i.e., the sum of the energies of the pump (U_p) and probe (U_{pr}) lasers. Since the probe is weaker than the pump, we let $U \sim U_p$. From temporal coupled mode theory, the expression for U_p is given by $U_p = f_p P_p$, where f_p is defined as [2]:

$$f_p = \frac{\gamma_e}{\left(\omega_{p,0}(1+\delta\omega) - \omega_p\right)^2 + \gamma_{\text{tot}}^2}, \tag{3}$$

in which γ_{tot} is the total loss rate of photons from the cavity, γ_e the extrinsic loss in the bus waveguide, and $\delta\omega$ the normalized frequency shift imparted by thermal and free carrier dispersion. The latter is given by $\delta\omega = -\frac{\Gamma}{n}\left(\sigma_{\text{FCD}}\Delta N + \sigma_{\text{TOE}}\Delta T\right)$, where Γ is the modal confinement factor, n the refractive index of the waveguide core material, σ_{FCD} the FCD coefficient, σ_{TOE} the thermo optic coefficient and ΔT the differential temperature of the MR with respect to the cold cavity condition. An similar expression holds for f_{pr}, which defines $U_{pr} = f_{pr} P_{pr}$. Note that Eq.(3) assumes that the MR internal energy adiabatically follows the input power variations P_p. In our case, this holds since P_p varies on the timescale of the free carrier lifetime $\tau_{\text{fc}} = 45\,\text{ns}$, while the photon lifetime is $\frac{2}{\gamma_{\text{tot}}} \sim 10\,\text{ps}$. Moreover, in our experiment the pump power varies much faster than the thermal decay constant of the MR [3], so that after an initial transient, the temperature reaches an equilibrium value $\overline{\Delta T}$. As discussed in Section 1.3 of the main text, for our MR this approximation holds up to $P_p = 6\,\text{dBm}$, after which thermal effects drive the cavity in an unstable regime and have to be accounted for in the dynamics. We now expand f_p^2 to the first order in ΔN around a reference value $\overline{\Delta N}$ as $f_p^2 \sim \overline{f_p^2} + \frac{df_p^2}{d\Delta N}\left(\Delta N - \overline{\Delta N}\right)$, where:

$$\frac{df_p^2}{d\Delta N} = \frac{4\frac{\Gamma\sigma_{\text{FCD}}}{n}\omega_{p,0}\gamma_e^2\left[\left(1+\delta\omega\right)\omega_{p,0} - \omega_p\right]^2}{\left[\left(\left(1+\delta\omega\right)\omega_{p,0} - \omega_p\right)^2 + \gamma_{\text{tot}}^2\right]^3}. \tag{4}$$

In deriving Eq.(4), we neglected the dependence of γ_{tot} on both ΔN and U, which is due respectively to FCA and TPA, since their contribution is small compared to FCD. If the pump power variations around the reference value $\overline{P}_{p,0}$, giving the equilibrium condition $\Delta N = \overline{\Delta N}$, are sufficiently small to justify the first order expansion of f_p^2, we can insert it into Eq.(2) to give:

$$\Delta N(t) = g_{\text{tpa}} \int_{-\infty}^{t} e^{\frac{t-\xi}{\tau_{\text{fc}}}} P_p^2(\xi) \left(\overline{f_p^2} + \frac{df_p^2}{d\Delta N}\left(\Delta N(\xi) - \overline{\Delta N}\right)\right) d\xi. \tag{5}$$

Since the probe and the pump lasers have similar detunings and linewidth γ_{tot}^{-1} with the corresponding MR resonances, we can also expand f_{pr} to the first order in ΔN. This allows to relate ΔN to the probe energy U_{pr} as:

$$\Delta N - \overline{\Delta N} = \left(P_{pr}\frac{df_{pr}}{d\Delta N}\right)^{-1}\left(U_{pr} - \overline{U_{pr}}\right). \tag{6}$$

By substituting Eq.(6) into Eq.(5) and rearranging terms, we obtain Eq.(1) of the main text, with the definitions:

$$c_0 = \gamma_e \left(\overline{U_{pr}} - \frac{df_{pr}}{d\Delta N} \overline{\Delta N} P_{pr} \right), \tag{7}$$

$$c_1 = \frac{df_{pr}}{d\Delta N} g_{\text{tpa}} \gamma_e \overline{f_p^2} P_{pr} \left(1 - \frac{1}{\overline{f_p^2}} \left(\frac{df_p^2}{d\Delta N} \right) \left(\frac{df_{pr}}{d\Delta N} \right)^{-1} \frac{\overline{U_{pr}}}{P_{pr}} \right), \tag{8}$$

$$c_2 = g_{\text{tpa}} \gamma_e \frac{df_p^2}{d\Delta N}. \tag{9}$$

By taking the values of the varius coefficients from [3], and by using $P_{pr} = 0.5\,\text{mW}$, one finds $c_1 \sim 2430\,\text{fJ}^{-1}$ and $c_2 \overline{U_{pr}} \sim -c_1$.

B. RELATIVE MAGNITUDE BETWEEN KERR AND FREE CARRIER EFFECTS

Using the same notation of the Supplementary material Section A, the normalized resonance shift $\delta\omega$ imparted by Kerr and FCD are given respectively by $\delta\omega_{\text{FCD}} = -\frac{\Gamma}{n}\sigma_{\text{FCD}}\Delta N$ and $\delta\omega_{\text{Kerr}} = -\frac{cn_2 U_p}{n^2 V}$ [4], where n_2 is the nonlinear refractive index of silicon, c the speed of light and V the volume of the MR. We can calculate ΔN as the steady state solution of Eq.(1) as $\Delta N = \tau_{\text{fc}} g_{\text{tpa}} U_p^2$, that combined to Eq.(3) yields the following expression for $\delta\omega_{\text{fc}}$:

$$\delta\omega_{\text{fc}} = -\frac{\sigma_{\text{FCD}} \tau_{\text{fc}} g_{\text{tpa}} q^2(\omega_p, \omega_{p,0}) Q^2 P_p^2}{16 \omega_p^2 n}, \tag{10}$$

where $q(\omega_p, \omega_{p,0})$ is defined as:

$$q(\omega_p, \omega_{p,0}) = \left(\left(\frac{\omega_{p,0}}{\gamma_{\text{tot}}} \left(1 + \delta\omega - \frac{\omega_p}{\omega_{p,0}} \right) \right)^2 + 1 \right)^{-1}. \tag{11}$$

For simplicity, we assumed $\gamma_{\text{tot}} \sim 2\gamma_e$, which in our case is justified by the fact that $Q_i = \frac{\omega}{\gamma_i} \gg Q_e$ (see Section 1 of the main text). Similarly, the expression for $\delta\omega_{\text{Kerr}}$ is given by:

$$\delta\omega_{\text{Kerr}} = -\frac{cn_2 q(\omega_p, \omega_{p,0}) Q P_p}{4 \omega_p n^2 V}. \tag{12}$$

We can now calculate the ratio between the FCD and the Kerr frequency shift as:

$$\frac{\delta\omega_{\text{fc}}}{\delta\omega_{\text{Kerr}}} = \frac{\sigma_{\text{FCD}} \tau_{\text{fc}} g_{\text{tpa}} q(\omega_p, \omega_{p,0}) n V Q P_p}{4 \omega_p c n_2}. \tag{13}$$

By inserting the values of the parameters in [3], and using $\sigma_{\text{FCD}} = -4 \times 10^{-27} \text{m}^3$ [5], $P_p = 5\,\text{mW}$ and $q = 0.95$ (which corresponds to the experimental pump detuning of $-7.5\,\text{GHz}$) we obtain $\frac{\delta\omega_{\text{fc}}}{\delta\omega_{\text{Kerr}}} \sim 200$. We expect the actual ratio to be slightly lower due to the degradation of the quality factor caused by TPA and FCA, which however does not alter the claim that FCD imparts a resonance shift which is two orders of magnitude higher than the one of the Kerr.

C. CALCULATION OF THE SIGNAL TO NOISE RATIO

In the following, we detail how the Signal to Noise Ratio (SNR) is estimated in case of the Iris flower classification task. The procedure, shown in Fig.1(a), can be divided in the following steps:

1. Step 1: The probe signal intensity is detected by a photodiode and recorded by an oscilloscope. Each flower sample is made by 50 virtual nodes of temporal duration 1/B, where B is the bitrate. The deadtime between different flower samples is set to 100 ns, and is discarded in the SNR analysis. The oscilloscope sampling frequency is 5 GHz, which corresponds to 250 sample points for each virtual node. In a preliminary stage, the data is smoothed using a moving average filter involving SP adjacent points. We collected 150 flower samples and stacked all the virtual nodes into the same dataset.

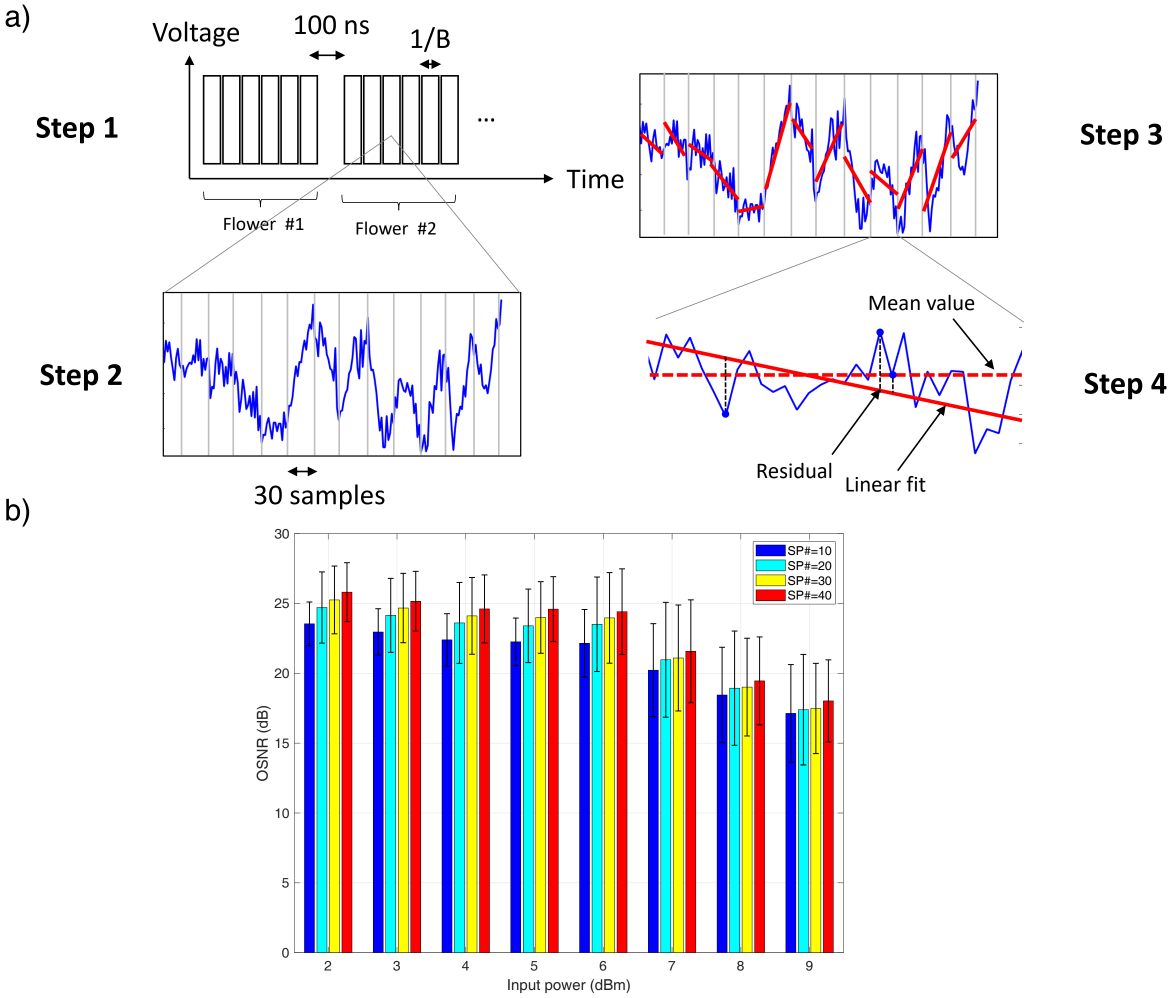

FIG. 1. (a) The four sequential steps involved in the calculation of the SNR in case of the task of Iris flower classification. (b) OSNR as a function of the input pump power and the number of smoothing points SP implemented in the moving average filter.

2. Step 2: Each virtual node is further divided into several disjoint sets made by 30 sampling points each.

3. Step 3: A linear fit is executed for each set. The slope and the intercept are determined by minimizing the sum of the residuals.

4. Step 4: A local SNR is evaluated for each set using the formula $\mathrm{SNR} = 10\log\frac{\langle V^2\rangle}{\langle N^2\rangle}$ where V are the voltage levels within each set and N the residuals. The average optical SNR (OSNR) is calculated among all the sets to yield the value reported in the main manuscript. We define the OSNR as SNR/2, since the SNR is referred to the electrical power ($\propto V^2$), while the OSNR is referred to the optical power ($\propto V$).

Steps from 1 to 4 are repeated for different values of $\mathrm{SP} = \{10, 20, 30, 40\}$, and for different values of the optical pump power. A summary of the obtained OSNR is reported in Fig.1(b). The calculation of the SNR for the XOR task follows the very same steps.

[1] T. J. Johnson, M. Borselli, and O. Painter, Self-induced optical modulation of the transmission through a high-q silicon microdisk resonator, Optics Express **14**, 817 (2006).
[2] S. Biasi, F. Ramiro-Manzano, F. Turri, P.-E. Larre, M. Ghulinyan, I. Carusotto, and L. Pavesi, Hermitian and non-hermitian mode coupling in a microdisk resonator due to stochastic surface roughness scattering, IEEE Photonics Journal **11**, 1 (2018).

[3] M. Borghi, D. Bazzanella, M. Mancinelli, and L. Pavesi, On the modeling of thermal and free carrier nonlinearities in silicon on insulator microring resonators, arXiv preprint:2012.05989 (2020).

[4] M. Borghi, *Linear, nonlinear and quantum optics in Silicon Photonics*, Ph.D. thesis, University of Trento (2016).

[5] Q. Lin, O. J. Painter, and G. P. Agrawal, Nonlinear optical phenomena in silicon waveguides: modeling and applications, Optics Express **15**, 16604 (2007).
